# Psychometric Curve and Behavioral Strategies for Whisker-Based Texture Discrimination in Rats

**DOI:** 10.1371/journal.pone.0020437

**Published:** 2011-06-01

**Authors:** Takeshi Morita, Heejae Kang, Jason Wolfe, Shantanu P. Jadhav, Daniel E. Feldman

**Affiliations:** Department of Molecular and Cellular Biology and Helen Wills Neuroscience Institute, University of California, Berkeley, California, United States of America; University of New South Wales, Australia

## Abstract

The rodent whisker system is a major model for understanding neural mechanisms for tactile sensation of surface texture (roughness). Rats discriminate surface texture using the whiskers, and several theories exist for how texture information is physically sensed by the long, moveable macrovibrissae and encoded in spiking of neurons in somatosensory cortex. However, evaluating these theories requires a psychometric curve for texture discrimination, which is lacking. Here we trained rats to discriminate rough vs. fine sandpapers and grooved vs. smooth surfaces. Rats intermixed trials at macrovibrissa contact distance (nose >2 mm from surface) with trials at shorter distance (nose <2 mm from surface). Macrovibrissae were required for distant contact trials, while microvibrissae and non-whisker tactile cues were used for short distance trials. A psychometric curve was measured for macrovibrissa-based sandpaper texture discrimination. Rats discriminated rough P150 from smoother P180, P280, and P400 sandpaper (100, 82, 52, and 35 µm mean grit size, respectively). Use of olfactory, visual, and auditory cues was ruled out. This is the highest reported resolution for rodent texture discrimination, and constrains models of neural coding of texture information.

## Introduction

Rodent whiskers are active tactile detectors that guide sensory and exploratory behavior [1], [2]. Two functionally distinct whisker systems—the long, moveable macrovibrissae and shorter, nonmoveable microvibrissae—are conserved across species [3], [4], [5], [6]. Rats use macro- and microvibrissae to detect a variety of tactile features of their environment, including object position [7], [8], [9], [10], shape [4], [11], [12], aperture and gap width [13], [14], [15], and surface texture (synonymous with roughness in the whisker literature) [1], [16], [17], [18], [19], [20], [21], [22]. Rats are capable of precise texture discrimination using the whiskers, including discrimination of aluminum surfaces with 1.00 vs. 1.06 mm-spaced grooves and sandpapers with 100 vs. 201 µm mean grit diameter [17], [20]. However, precisely how whiskers detect texture and other surface features is not yet understood.

Several potential coding mechanisms have been proposed for texture detection by the whiskers [23], [24], [25]. In one model, texture is related to the mean speed or total power of surface-induced vibrations in the macrovibrissae [26], [27] encoded in mean firing rate of somatosensory cortex (S1) neurons [22], [26]. In another, texture is encoded by the rate or magnitude of high-velocity, high-acceleration ‘slip-and-stick’ motion events in macrovibrissae [26], [28], [29] which drive transient firing correlations in S1 [30]. Alternatively, texture may be encoded by the relative amplitude of vibrations across different-length whiskers, which have different mechanical resonance frequencies [21], [31].

Quantitative evaluation of these models requires comparison to a psychometric curve for texture discrimination, as has been done for roughness perception in primate fingertips [32], [33], [34] and for detection and discrimination of vibrotactile stimuli in the whisker system [35], [36]. However, no quantitative psychometric curve for whisker-based texture discrimination has been reported. In addition, while sensory coding models focus on the macrovibrissae, it is not rigorously established whether the primary sensors for high-acuity texture discrimination are the macro- or microvibrissae, because most texture discrimination studies involved contact with both [e.g., 17,18], and microvibrissae are capable of mediating form discrimination [4]. Here, we tested the ability of macro- and microvibrissae to mediate texture discrimination, and measured a psychometric curve for macrovibrissa-based discrimination of sandpaper surfaces, in order to constrain models of neural coding of texture.

Results showed that rats sense surface texture with at least two distinct behavioral strategies: macrovibrissae-based discrimination, which is performed with the nose >2 mm from surfaces, and microvibrissae- and non-whisker tactile based discrimination, which is performed with the nose 0–2 mm from surfaces. We report a psychometric curve for macrovibrissae-based discrimination of sandpaper textures, which reveals the highest known resolution for whisker-based texture discrimination.

## Materials and Methods

All procedures were approved by the UC Berkeley and UCSD Institutional Animal Care and Use Committees (protocols R309 and S01040R, respectively). 19 female Long-Evans rats (150 g at start of training) underwent texture discrimination training. 7 of these rats learned the task to criterion and used whiskers, not paws, for surface palpation. Data from these 7 animals are reported here. Rats were housed in groups of 2–3 littermates. Rats were typically given a single 30–45 min training session daily, 5 days per week, during the light component of the 12 hr light/dark cycle. To motivate training, water was restricted 22 hrs prior to training, and was available as behavioral reward during conditioning and during a 0.5–1 hr free drinking period after each training session. Food was available ad lib in the home cage. Rats gained weight normally and displayed normal behavior throughout the entire training period (3–6 months).

### Behavioral training

Training was performed in one of two computer-controlled operant conditioning chambers that implemented a 2-alternative forced choice texture discrimination task. The first chamber was used for sandpaper texture discrimination (Paradigm 1.) The second was used for discrimination of smooth vs. grooved surfaces (Paradigm 2). Each rat was trained in only one chamber.

### Sandpaper discrimination (Paradigm 1)

**Figure 1:**
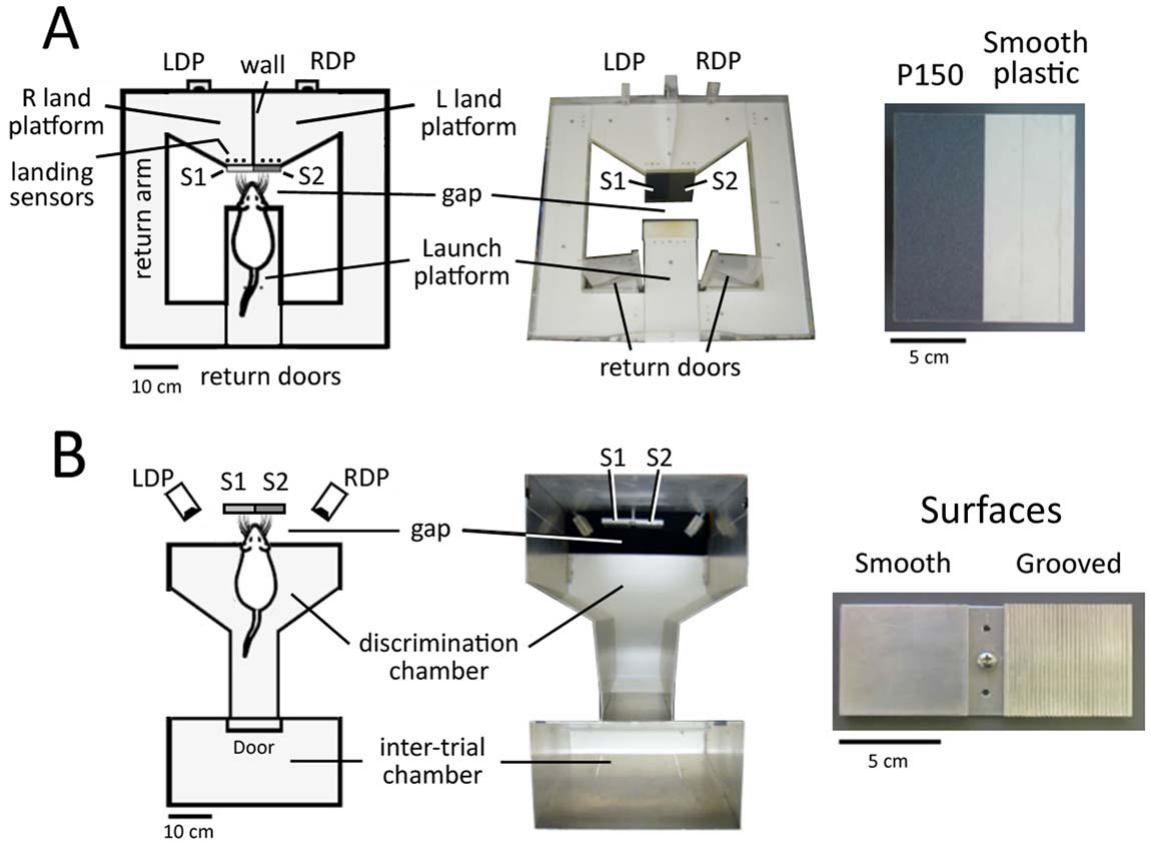
Texture discrimination chambers. **A**, Sandpaper discrimination chamber for paradigm 1. S1 and S2, sandpaper surfaces. LDP and RDP, left and right drink ports. Initial training was performed with rough (P150) sandpaper vs. smooth plastic (pictured). S1 and S2 position was randomly interchanged between trials. **B**, Smooth-grooved discrimination chamber for paradigm 2. S1 and S2, smooth or grooved surfaces. Left-right position of smooth and grooved surfaces was interchanged randomly between trials.

The chamber (**Fig. 1A**) contained an elevated central launch platform separated from two landing platforms by a variable-sized gap. Platforms were plexiglass with a low (1-cm) wall, except for the front of the launch platform (facing the gap), which had no wall and a high-grip Velcro floor. Facing the launch platform within the gap were two 6×18 cm strips of commercial sandpaper (3M Corporation; grades P120 [roughest] to P1200 [smoothest]; P grit values reflect the ISO 6344 industrial standard) mounted vertically on a 12×18 cm acrylic plate. The sandpaper samples were mounted side-by-side with no gap between them. A computer-controlled stepper motor rotated the sandpaper plate to switch the left-right position of the sandpapers. Behind each sandpaper was a landing platform that contained an infrared LED-phototransistor landing sensor and a drink port. The sandpaper panel extended 0–1 cm vertically above the height of the landing platforms. Rats were trained to reach across the gap, palpate the textures with the whiskers and select the rougher of the 2 sandpapers by jumping across the gap to the landing platform behind the rougher sandpaper, similar to [16] and [18].

Rats self-initiated each trial by moving to the front edge of the launch platform and reaching across the gap with their whiskers to palpate the textures. After a variable period of palpation, rats jumped the gap to land on the left or right landing platform. Rats were rewarded (0.05–0.1 mL water) for choosing the landing platform behind the rougher (S+) sandpaper, while choosing the S- landing platform triggered a time out tone. After each trial, rats returned to the launch platform either via left and right return arms or by jumping back over the gap. The right-left position of the sandpapers was randomly changed between trials while the rat was either in the return arm or at the back of the launch platform. The motor was rotated 90° in one direction and then either 90° in the same direction (to exchange texture positions), or 90° in the opposite direction (to maintain the same texture positions between trials). This eliminated auditory cues for texture exchange. Stimulus rotation, drink port choice and reward delivery were controlled and recorded by custom routines in LabView (National Instruments) and Igor (Wavemetrics). Data analysis was performed in Matlab (Mathworks, Natick MA).

Training was performed in stages. In Stage 1, rats were acclimated to handling and to the training cage (15 min per day, for 1 week). In Stage 2, rats were trained in complete darkness to drink from a single reward port placed at the front edge of the launch platform (5–7 days). In Stage 3, two very distinct textures (rough P120 or P150 sandpaper [S+] and smooth plastic [S−]) were introduced along with drink ports on the landing platforms, and the full reward contingency was implemented. Rats had to self-initiate trials and jump across a 10 cm-gap to receive a reward from the platform behind the S+ texture. Incorrect choice (S−) triggered a time out (4 seconds, accompanied by the time out tone). Initially, the left-right position of S+ and S- textures were varied in blocks of 5–20 trials to facilitate learning; blocks were gradually reduced in size and then eliminated, with S+ and S− position randomly assigned on each trial. In addition, sandpaper or smooth plastic strips were initially placed on the upper edge of the rotating panel, to function as confirmatory texture cues available if rats stepped on the sandpaper panel top during gap crossing. Once discrimination was learned, these strips and the top 2 mm of sandpaper were removed from the panels, so that no texture cue was obtained by stepping on the panel top during gap crossing. Stage 3 training was performed daily for 0.5–2 months until rats learned the task (see Results). In the final days of Stage 3, the gap was increased to 10–13 cm to discourage microvibrissa or nose contact.

### Smooth vs. grooved surface discrimination (Paradigm 2)

The conditioning chamber consisted of an elevated inter-trial chamber (25×35 cm) and a discrimination chamber (50×35 cm), separated by a door (**Fig. 1B**). Chambers had plexiglas floors and 35-cm aluminum walls. Trials started by placing the rat in the inter-trial chamber. The door was manually opened, and rats entered the discrimination chamber, where they reached with their head and whiskers across a gap in the floor (9.0–9.5 cm) to sample two square aluminum surfaces (6×6 cm each, placed side by side, oriented vertically). One surface was smooth aluminum (S−) and the other was milled with vertical grooves (1 mm groove width and spacing, 0.5 mm groove depth) (S+). Surfaces were mounted on a rotating stepper motor, which allowed the right-left position of S+ and S− to be randomly interchanged between trials. As in Paradigm 1, rotation was performed in two 90° steps to eliminate auditory cues for texture exchange.

After palpating the surfaces, rats could place their nose in the left or right drink port located immediately lateral to the surfaces, where an infrared emitter-detector system (890 nm) detected nose entry. Selection of the drink port adjacent to the S+ texture (within 20 sec of sampling the textures) delivered 0.1 mL water via solenoid valve. Nose poke at the S− port triggered a timeout tone (2 sec). Rats were then manually ushered back to the inter-trial chamber, while the stimuli were randomly rotated in preparation for the next trial. The chamber was controlled by routines in Lab View (National Instruments).

Training was performed in stages. In Stage 1, rats were acclimated to the training cage as in Paradigm 1. In Stage 2A, water restriction began and rats were trained to drink water dispensed manually from a single drink port with no gap (1–2 days). In Stage 2B, rats were trained to nose poke in the drink port to obtain reward (2–3 days). In Stage 3, textures and both drink ports were introduced along with a small 2–3 cm gap, and reward was dispensed only for correct S+ choices. Approximately 3 weeks of Stage 3 training were required to learn the contingency. The gap was widened gradually to 9.0–9.5 cm, to promote sampling with the macrovibrissae. However, even at this gap distance, the nose touched the surface in many trials (see Results). Stages 1–3 were performed in dim room light. Once rats learned the contingency (late Stage 3) training was performed entirely in the dark, with the experimenter wearing infrared goggles.

### Video analysis

Whisker and head movements during texture sampling were recorded by a video camera (30 noninterlaced frames/sec) above or below the gap, using infrared LED illumination Frame-by-frame manual analysis enabled classification of trials into those in which the nose approached <2 mm from the surface (short distance trials) vs. trials in which the nose remained ≥2 mm from the texture (long distance trials). We discarded rare trials in which rats did not make whisker contact or contacted surfaces with the paw before behavioral choice.

### Whisker trimming

For some experiments, microvibrissae or macrovibrissae were trimmed. Trimming was performed under transient isoflurane anesthesia (3–4% in 2 L/min oxygen), and whiskers were cut with scissors to the level of the fur. Trimming was repeated once every 2–3 days to prevent whisker regrowth. The macrovibrissae were defined as A and B whisker rows, arcs 1–4 of the C-E whisker rows, and the Greek (straddler) whiskers. The microvibrissae were defined as the more rostral (arc 5+) whiskers in the C–E rows, all whiskers in the F–J rows, and additional small hairs on the lip (Brecht et al., 1997).

### Psychometric curve for sandpaper texture discrimination

A psychometric curve for texture discrimination was constructed by holding the S+ sandpaper constant, and varying the S- sandpaper in a block design. The constant S+ stimulus was termed the ‘base’ stimulus, and the varying S- stimulus was termed the ‘test’ stimulus. In each block, a single test sandpaper was used, and ∼25 trials were obtained to measure discrimination performance. Three blocks using different test stimuli were presented on each day, by manually switching sandpaper texture panels between blocks. Block order was chosen to interleave different test sandpapers in a counterbalanced manner, while maintaining similar day-to-day difficulty levels. In some blocks, the test stimulus was identical to the base stimulus, which should result in 50% correct choices, and was used to confirm that texture was guiding behavioral choice. Accuracy was determined for each test sandpaper block in which the animal performed ≥10 trials. (Blocks with <10 trials were rare and were associated with significantly worse performance, which may indicate low motivation or attention.)

For three animals, the S+ ‘base’ stimulus was the rough P150 sandpaper, and ‘test’ stimuli were varying smoother sandpapers. Thus, one day's blocks might be P1500 vs. P150 (Block 1, easy), P400 vs. P150 (Block 2, moderate), and P180 vs. P150 (Block 3, very difficult). The next day would use different S− stimuli, in a counterbalanced order, with similar overall daily difficulty. For one animal, an additional psychophysical discrimination curve was obtained using smooth P1500 sandpaper as the ‘base’ stimulus. This was done by retraining the rat that P1500 was the rewarded S+ stimulus (24 days of training). The psychometric curve was then measured with P1500 as the S+ ‘base’ stimulus, and variety of rougher sandpapers as S−.

### Olfactory controls

To test whether rats differentiated smooth vs. grooved aluminum surfaces by deposited olfactory cues, trial blocks were performed in which we wiped each surface with 70% ethanol and allowed it to completely dry before use. This will reduce but not eliminate odors. We compared performance between wipe and non-wipe trial blocks. Two olfactory controls were performed for sandpaper discrimination. To test for use of deposited olfactory cues, we replaced sandpaper discriminanda with new sandpaper samples on some trials. To test for use intrinsic olfactory cues in the sandpaper glues or paper backings, we compared performance on trials with the sandpaper grain side facing the rat vs. trials with the sandpaper reversed so that the paper backing faced the rat. If rats performed discrimination based on intrinsic olfactory cues, performance should remain intact during sandpaper reversal.

## Results

### Rats learn both sandpaper discrimination and smooth-grooved surface discrimination

**Figure 2:**
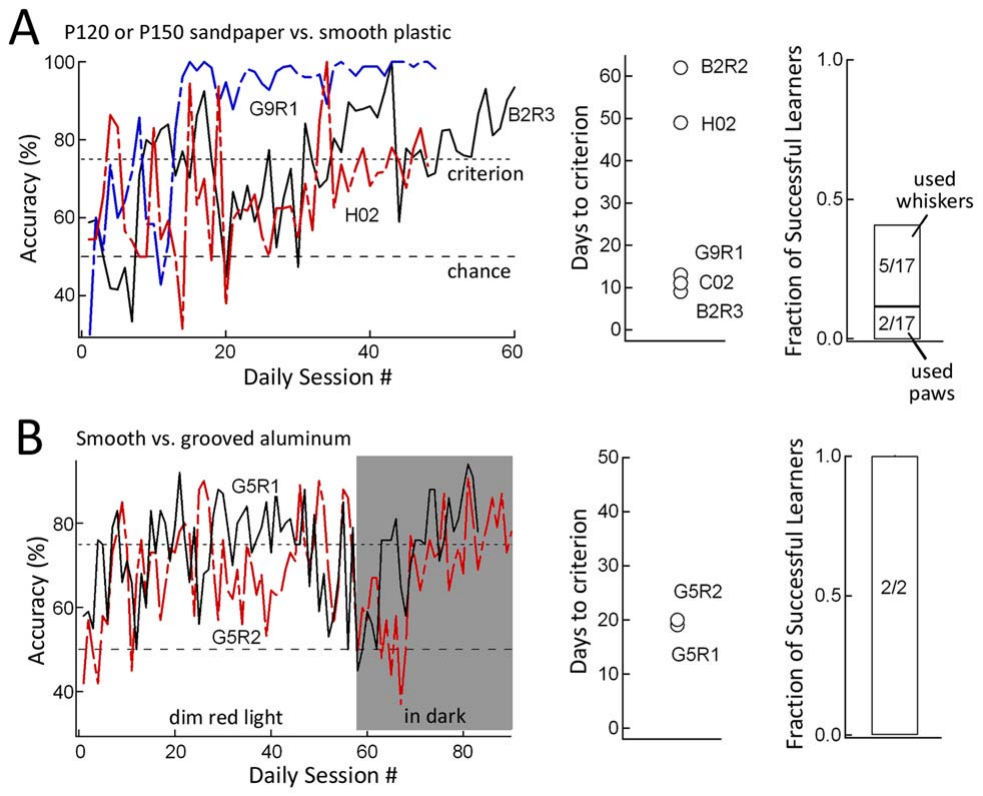
Learning curves for sandpaper and smooth vs. grooved discrimination. **A**, Sandpaper discrimination. Left, learning curve for 3 example rats. Day is is start of stimulus-reward contingency (Stage 3). Middle, Number of daily training sessions to reach criterion performance, for each successful learner. Right, Fraction of successful learners. **B**, Smooth-grooved discrimination. Left, learning curve for 2 rats. Rats were initially trained in dim light, and then switched to darkness. Middle, number of training sessions to reach criterion performance. Right, Fraction of successful learners.

17 rats were trained in Paradigm 1 on the sandpaper discrimination task. After initial training to drink from the reward port (Stages 1–2, see Methods), a rough sandpaper (P120 or P150) (S+) and a smooth plastic film (S−) were presented side-by-side in the gap between launch and landing platforms. Left-right position of these surfaces varied randomly between trials. Rats were rewarded for jumping to the landing platform behind the S+ (rough) surface, while jumping to the S− (smooth) landing platform triggered a time out tone and no reward. Training occurred in the dark. The gap was initially small (1 cm), and was gradually increased to a final size of 10–13 cm as rats learned the discrimination, in order to promote the use of whiskers for texture palpation. 5/17 rats (G9R1, B2R2, B2R3, C02, H02) showed gradual improvement in discrimination accuracy, reaching criterion performance (≥75% performance for 3 consecutive days) within 15–36 days (**Fig. 2A**). In these rats, video analysis showed they explored the surface using the whiskers rather than paws or nose. Two more rats learned to criterion, but video analysis showed surface exploration with the paws (G11R1 and G11R3). The remaining rats performed very few trials per day (reflecting a hesitancy to jump the gap in the dark) or performed sufficient trials but did not reach criterion performance within 70 days.

Two additional rats (G5R1 and G5R2) were trained in Paradigm 2 to discriminate between an aluminum plate milled with 1-mm spaced grooves (S+) and a smooth aluminum plate (S−). Initial training (Stage 1 to beginning of Stage 3, see Methods) was in low room lighting. Both rats showed gradually improvement in discrimination accuracy, reaching criterion performance (≥75% correct for 3 consecutive days) after 19 and 20 days (**Fig. 2B**). To eliminate visual cues, these rats were then trained in complete darkness, and stable performance at or above the 75% criterion was reattained in 6–26 days. During this same period, gap width was increased to 9.5 cm (G5R1) and 9.0 cm (G5R2) to promote use of the whiskers for texture palpation.

### Two behavioral strategies for texture discrimination

**Figure 3:**
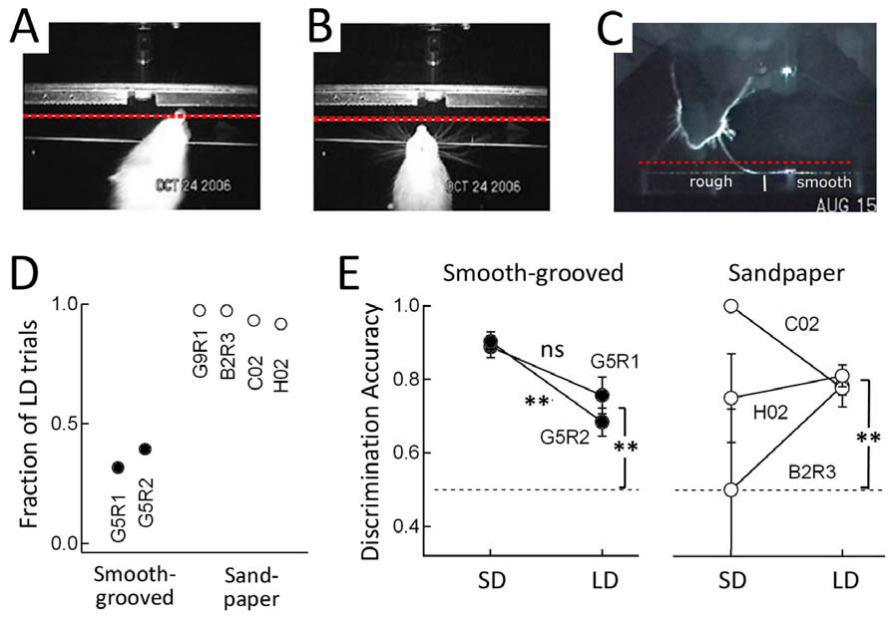
Short-distance (SD) and long-distance (LD) sampling strategies for texture discrimination. **A and B**) Examples of SD sampling (A) and LD sampling (B) during smooth-grooved discrimination (Rat G5R2). Video frames were taken from underneath the rat. Dashed line, 2 mm from surface. LD trials were defined as trials in which the nose remained >2 mm from surface during all frames of sampling. **C**) Example of LD sampling during sandpaper discrimination. Dashed line, 2 mm from surface. **D**) Fraction of LD trials for rats performing smooth-grooved and sandpaper discrimination. **E**) Discrimination accuracy during LD and SD trials for smooth-grooved discrimination (left) and sandpaper discrimination (right, compiled for P150 vs. P1500 or P1200 discrimination). Asterisks, significant differences from chance (binomial exact test) or between grouops (two-sample equal proportion test). ns, no significant difference.

Videography was used to monitor head position relative to the textures in rats performing smooth-grooved and sandpaper discrimination. We distinguished two types of trials: 1) long-distance (LD) trials in which the nose remained ≥2 mm from the texture throughout the trial; 2) short-distance (SD) trials in which the nose approached within <2 mm from the texture. Examples of these two trial types are shown in **Fig. 3A–C**. Both of these trial types involved a distinct sampling period in which the nose or whiskers lingered over the texture before a ballistic head/body movement was made to steer the head toward the selected reward port, as in [22]. Postmortem measurements in 2 adult female rats showed that macrovibrissae extend >2 mm in front of the nose at maximal protraction, while the microvibrissae do not (**Table 1**). Therefore, LD trials (nose ≥2 mm from the texture) are likely to involve macrovibrissae contact with surfaces, while SD trials (nose <2 mm from the texture) may involve nose, microvibrissae, and/or macrovibrissae contact.

**Table 1:** Maximal forward extend of whiskers relative to the nose.

Whiskers	D (mm)	N
Greek (macro)	39.0±2.3	7
Arc 1 (macro)	31.4±1.4	8
Arc 2 (macro)	22.1±1.6	8
Arc 3 (macro)	12.5±1.6	8
Arc 4 (macro)	7.4±1.3	6
Arc 5 (micro)	1.9±0.9	5
Arc 6–7 micro, F–G row micro	−1.1±0.5	18

D is mean rostrocaudal distance (± SEM) between whisker tip and rostral tip of nose, with whisker manually held in its maximal protracted position. Positive values are whisker tip rostral to nose. N, number of whiskers measured (n = 2 rats).

In smooth-grooved discrimination, where the gap distance was moderate (9–9.5 cm), rats performed 60–70% SD trials and 30–40% LD trials (**Fig. 3D**). Both rats G5R1 and G5R2 successfully discriminated smooth vs. grooved surfaces on LD trials (0.76±0.05 and 0.68±0.04 fraction correct, n = 120 and 138 trials, error is SEM across multiple behavioral sessions). This performance was significantly above chance (p<2.5×10^−6^, binomial exact test). Rats also discriminated on SD trials (0.88±0.03 and 0.90±0.03, n = 258 and 212 trials, p<2.2×10^−16^). SD performance was significantly better than LD performance (p<0.002, binomial test) (**Fig. 3E**).

In sandpaper discrimination, a larger gap distance was used (10–13 cm), which strongly discouraged SD trials, and rats performed 92–97% LD trials (**Fig. 3D**). Discrimination accuracy was calculated for LD and SD trials for 3 rats (B2R3, C02, H02) during discrimination of P150 (rough) vs. P1500 or P1200 (very smooth) sandpapers. Discrimination accuracy on LD trials was 0.78±0.03, 0.78±0.05, and 0.81±0.03 for each rat (n = 197–359 trials, all animals significantly above chance, p<1.4×10^−8^, binomial exact test) (**Fig. 3E**). Performance on the few SD trials was 0.50±0.22, 0.75±0.12, and 1.0±0.0 for these animals (n = 10–18 trials, insufficient trials to determine whether performance was above chance). Thus, rats successfully discriminated surfaces at both long- and short distances, but sandpaper discrimination was performed nearly exclusively at long distance.

### Dependence on macro- and microvibrissae

**Figure 4:**
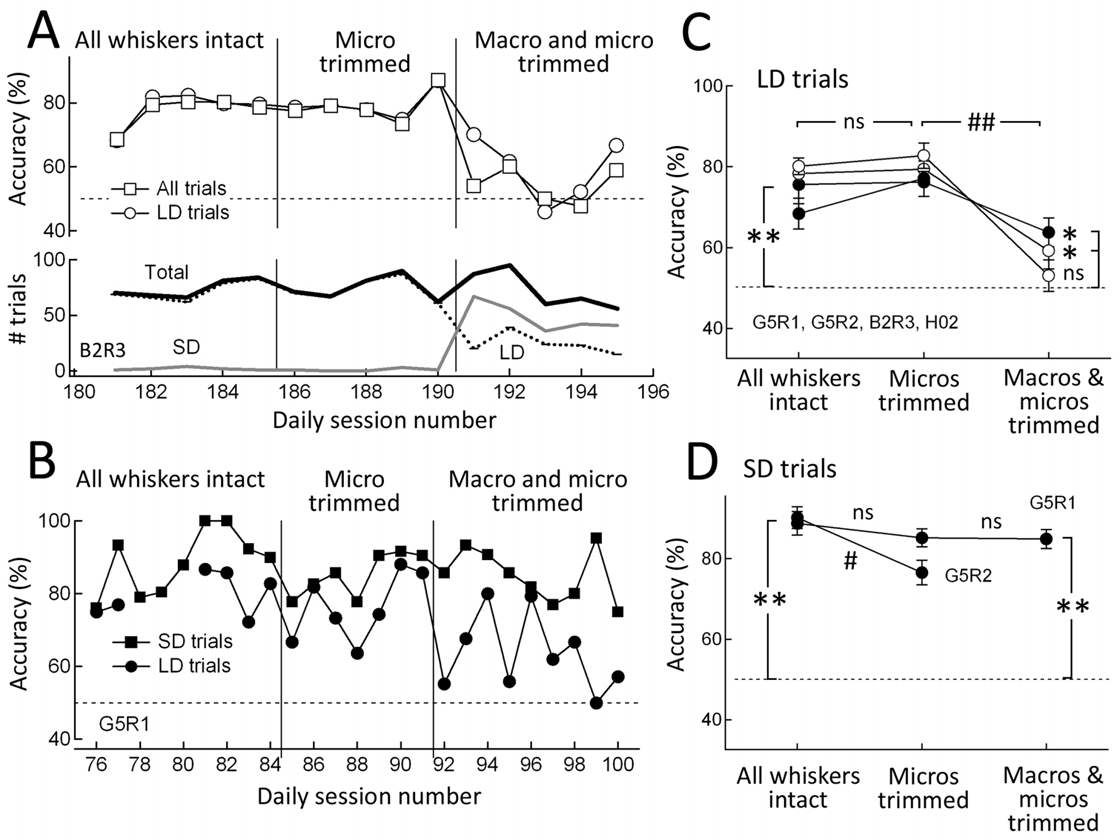
Dependence of SD and LD texture discrimination on microvibrissae vs. macrovibrissae. **A**, Performance of rat B2R3 on P120 vs. P1500 sandpaper discrimination during sequential trim of micro- and macrovibrissae. Each point is one behavioral session. Bottom: number of SD, LD and total trials per session. **B**, Performance of rat G5R1 on smooth-grooved discrimination. There were an average of 23 SD and 25 LD trials per day. Gaps indicate days with too few LD trials to calculate mean accuracy. **C**, Mean discrimination accuracy for LD trials for all rats in whisker trim experiments. Filled symbols are smooth-grooved discrimination. Open symbols are P120 or P150 (very rough) vs. P800 or P1500 (smooth) sandpaper discrimination. Each point is mean of 6–21 sessions (83–802 LD trials). Error bars are SEM across daily sessions. * and **, p<0.003 and p<2×10^−6^ relative to chance (binomial exact test). #, p<0.003 difference between groups (2-sample equal proportion test). ns, not significant. **D**, Mean discrimination accuracy for SD trials for rats performing smooth-grooved discrimination. Each point is the mean of 7–21 sessions (116–385 SD trials). SD trials in sandpaper discrimination were too few to be analyzed.

To determine whether macrovibrissae or microvibrissae are used for texture discrimination under our conditions, we tracked discrimination performance while sequentially trimming microvibrissae and then macrovibrissae (**Fig. 4**). In sandpaper discrimination, microvibrissa trim did not affect accuracy on LD trials, but subsequent macrovibrissa trim decreased accuracy to chance for LD trials (and for all trials, since the vast majority of trials were LD trials). This is shown for one example rat (B2R3) performing P120 (rough) vs. P1500 (smooth) discrimination in **Fig. 4A**. In smooth-grooved discrimination, microvibrissa trim did not substantially affect accuracy on either LD or SD trials. Subsequent macrovibrissa trim significantly decreased LD performance to near-chance, but accuracy on SD trials was not affected. This is shown for one example rat (G5R1) in **Fig. 4B**. Population results across 2 rats performing P120 vs. P1500 and P150 vs. P800 sandpaper discrimination and 2 rats performing smooth-grooved discrimination are shown in **Fig 4C, D**. These results show that LD discrimination required macrovibrissae, but not microvibrissae. In contrast, SD discrimination was not dependent on macrovibrissae, and surprisingly was only modestly dependent on microvibrissae (performance was partially impaired by microvibrissa trim in 1 rat, but not another). Thus, rats use macrovibrissae for texture discrimination at long distance. At short distance, microvibrissae may provide one cue for texture, but additional non-microvibrissa cues must also be used, perhaps including direct palpation of the surface with the skin of the nose or upper lip.

LD and SD trials appeared to represent voluntary, alternative strategies for texture discrimination, because macrovibrissa trim caused some rats to increase the proportion of SD trials during sandpaper discrimination (e.g., **Fig. 4A**), and microvibrissa trim caused rats to increase the fraction of LD trials in smooth-grooved discrimination (G5R2: pretrim, 39%, micros trimmed: 69%; G5R1: pretrim, 58%, micros trimmed: 65%).

### Psychometric curve for sandpaper texture discrimination

A psychometric curve for sandpaper discrimination was obtained in 3 rats (B2R3, C02, H02) discriminating rough P150 sandpaper (rewarded, S+) from varying smoother sandpapers (unrewarded, S−). In each trial, the P150 sandpaper (termed the ‘base’ sandpaper) was presented along side a smoother ‘test’ sandpaper selected from P1500, P1200, P800, P400, P280, P180, and P150. Left-right position of test and base sandpapers varied randomly between trials. For rats C02 and H02, daily behavioral sessions were divided into blocks of ∼25 trials (each block used a single test sandpaper). 3 blocks were presented per day, with block order counterbalanced, and easy and difficult discriminations intermixed to achieve a similar overall difficulty each day. For blocks in which P150 was used as both test and base stimuli, chance performance is expected. 5–15 blocks were completed for each test sandpaper. For rat B2R3, each test sandpaper was tested in 4–10 consecutive full-day blocks, after which a new test sandpaper was introduced. The order was from easier to harder discriminations (test: P1500, then P1200, P800, and P400) and then back to easier discriminations (test: P1200, then P800 and P1500). Interspersed were single days in which P150 was used as both test and base stimuli. Discrimination accuracy was calculated across all trials (which included 92–97% LD trials and 3–7% SD trials).

**Figure 5:**
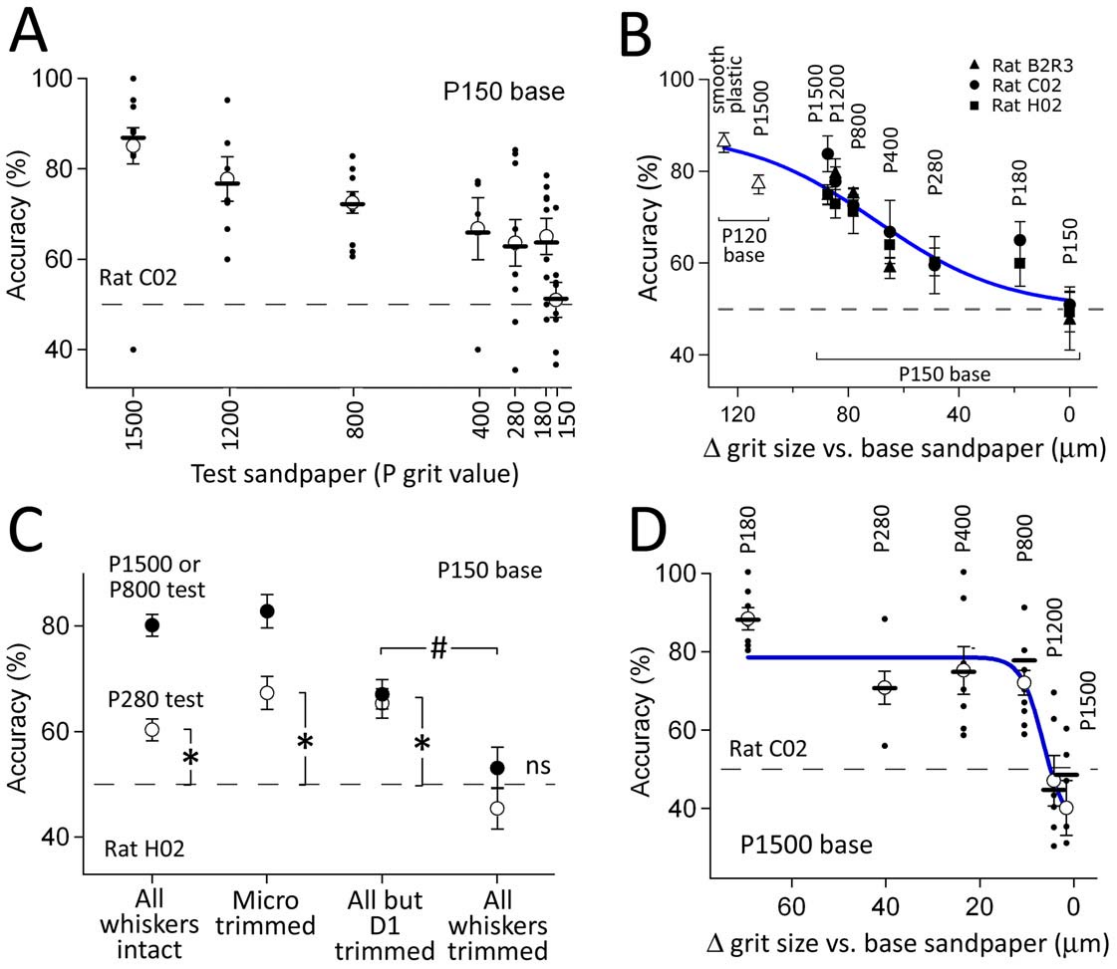
Psychometric curve for sandpaper texture discrimination. **A**, Performance of Rat C02 discriminating rough P150 sandpaper (base stimulus) from varying smoother (test) sandpapers. Each dot is performance on one daily block (∼25 trials). Open circles, mean accuracy across daily sessions ± SEM. Bars, cumulative accuracy (total correct trials/total trials). **B**, Mean performance of 3 rats for P150 base discrimination, and for one rat using P120 base. Bars are SEM across daily blocks (n = 4–15 blocks per data point). The x-axis is the difference in mean grit size between the test and base sandpapers. Test sandpaper identity is marked above each point. **C**, Effect of whisker trimming on difficult (P150 vs. P280) and easy (P150 vs. P1500 or P800) discriminations, for rat H02. **D**, High-acuity discrimination of a smooth P1500 sandpaper (base) from varying rougher (test) sandpapers. P1500 was the rewarded S+ stimulus.

Example performance for rat C02 is shown in **Fig. 5A**, and all 3 rats are shown in **Fig. 5B**. All rats performed P150 vs. P1500 discrimination at high accuracy (75–86% correct, 328–626 trials, significantly greater than chance, p<2.2×10^−6^, binomial exact test). Discrimination was also strong and significant for P150 vs. P1200 and P150 vs. P800. Remarkably, all 3 rats discriminated P150 vs. P400 sandpapers significantly above chance (58–64% correct, 167–380 trials, p<0.0006). Two rats (C02 and H02) were tested on P280 and P180 test sandpapers, and also discriminated these significantly above chance (60–64% correct, 234–312 trials, p<0.0004). In contrast, performance was at chance when test and base sandpapers were both P150 (49–51% correct, 178–242 trials, p > 0.37).

To relate discrimination performance to a physical surface feature, we plotted discrimination accuracy as a function of the difference in mean grain diameter between test and base sandpapers (125 µm [P120], 100 µm [P150], 82 µm [180], 52.2 µm [P280], 35 µm [P400], 21.8 µm [P800], 15.3 µm [P1200] and 12.6 µm [P1500]) (grain sizes from ISO 6344 industrial standard). The mean psychometric curve was fit with a logistic function (**Fig. 5B**). Thus, successful discrimination of P150 from P400 sandpaper (3 rats) and P150 from P280 and P180 sandpapers (2 rats) corresponds to discrimination of 65 µm, 48 µm, and 18 µm differences in mean grain size, respectively.

In one rat (H02), we confirmed that difficult sandpaper discrimination (P150 vs. P280) was dependent on macrovibrissae, but not microvibrissae, as shown above for easy (P150 vs. P1500 or P800) discriminations (**Fig. 5C**). P150 vs. P280 discrimination accuracy was unaffected by microvibrissa trim. To determine if a single macrovibrissa could mediate texture sensation, we then trimmed all whiskers except for D1 on each side of the face (**Fig. 3C** shows an image of performance with only D1 intact). This also did not reduce P150 vs. P280 discrimination accuracy (although it did partially reduce P150 vs. P1500 accuracy). Finally, we trimmed D1 so that no whiskers remained. This abolished P150 vs. P280 reduced performance to chance (45.4±4.5%, p = 0.9). Thus, difficult sandpaper discrimination required macrovibrissae but not microvibrissae, and could be performed with a single intact D1 whisker.

Finally, we tested in one rat (C02) the ability to distinguish a smooth P1500 sandpaper from varying rougher sandpapers (P180, 280, 400, 800, 1200). After completing the discrimination curve with P150 as base stimulus (**Fig. 5A**), we designated P1500 the rewarded (S+) stimulus, and P150 was the unrewarded (S−) stimulus. Retraining on this stimulus contingency took 24 days. We then measured a second psychometric curve in which P1500 sandpaper was distinguished from varying rougher sandpapers, presented in an interleaved, counterbalanced block structure. The rat showed high accuracy in discriminating P1500 from P180, 280, 400, and 800 test sandpapers (p<0.05, binomial exact test), while discrimination of P1200 vs. P1500 and P1500 vs. P1500 was at chance (**Fig. 5D**). The resulting logistic curve fit (blue) showed a rapid rise in discrimination ability between P1200 and P800 test stimuli. P1500 vs. P800 corresponds to significant discrimination of a 9 µm difference in mean grain size.

### Olfaction is not used for texture discrimination

**Figure 6:**
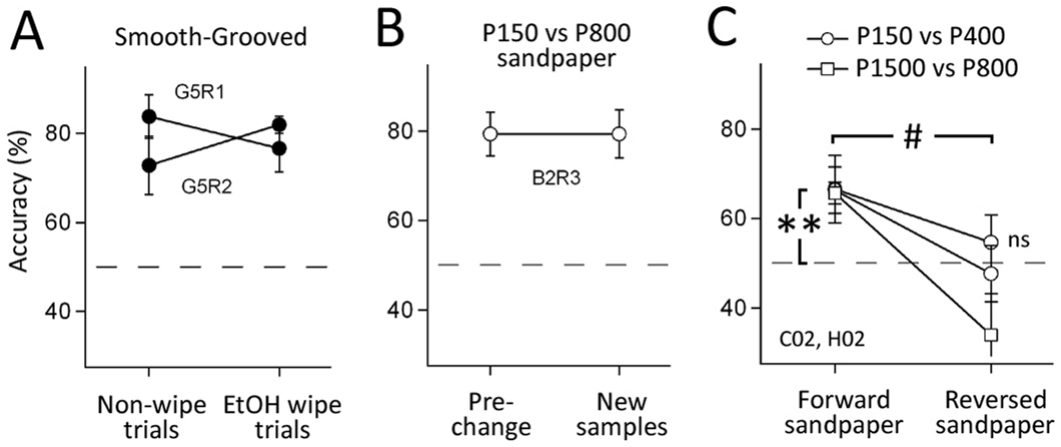
Olfactory cues do not mediate texture discrimination. **A**, Cleaning smooth-grooved surfaces with ethanol between trials (“EtOH wipe trials”) did not reduce discrimination performance relative to interleaved “non-wipe” trials. **B**, Exchanging P150 and P800 sandpaper samples that were used daily with new samples of each sandpaper did not alter discrimination accuracy. **C**, Reversing the sandpapers so that the paper backing, rather than the grit surface, faced the rat abolished discrimination. **, discrimination significantly greater than chance (p<0.0003). #, significantly less than the forward condition (p<0.04).

It is critical that rats use tactile cues, and not olfactory cues, to solve the texture discrimination task. We tested this in three experiments. First, in Rats G5R1 and G5R2 (smooth-grooved discrimination), we wiped the aluminum discriminanda with ethanol, and then allowed the surfaces to air-dry, before a subset of trials within each daily session. Ethanol wipe will disrupt volatile odorant cues that could have been deposited by the rat. Performance was not different on wipe- vs. non-wipe trials, indicating that deposited ethanol-sensitive odorants were not being used as cues for the discrimination (**Fig. 6A**). The second experiment was performed using rat B2R3 while it was discriminating between P150 and P800 sandpapers. We divided each daily training session into an epoch that used the standard sandpaper samples that had been used for many days, and a second epoch in which we replaced these with new, unused sandpaper samples. The goal was again to eliminate deposited olfactory cues. Performance was not different in pre-change vs. post-change epochs, indicating that deposited odors were not being used for discrimination (**Fig. 6B**).

Finally, to test whether rats in the sandpaper task performed discrimination based on intrinsic olfactory cues within the sandpapers (i.e., the different paper backings and glues used in manufacture of different sandpaper grades), we performed a ‘reversed sandpaper’ experiment using rats C02 and H02. In interleaved blocks, rats discriminated either P150 vs. P400 sandpaper or these same sandpapers mounted in reversed orientation (paper backing facing the rat) so that texture cues were eliminated but intrinsic olfactory cues remained. Both rats discriminated significantly above chance on the forward (normal) orientation (67 and 69%, 161 and 123 trials, p<1.4×10^−5^, binomial exact test), but discrimination was reduced to chance on the reversed orientation (46 and 56%, 146 and 92 trials, p> 0.12). Identical results were obtained for rat C02 discriminating P1500 (base) vs. P800 (test) in forward vs. reverse orientations (**Fig. 6C**). Thus, rats appeared not to use intrinsic olfactory cues to solve sandpaper texture discrimination.

## Discussion

### Two behavioral strategies for tactile discrimination

Our results show that rats can sense surface texture either using long-distance sampling (nose >2 mm from surface) or short-distance sampling (nose <2 mm from surface). With moderate gaps (as in the smooth-grooved discrimination task), rats used a mixture of long-distance and short-distance sampling; with larger gaps (as in the sandpaper discrimination task), rats almost exclusively used long-distance sampling. The smooth-grooved task allowed us to compare the whisker dependence of these sampling strategies. The sandpaper task allowed us to measure the resolution of texture discrimination during long-distance (macrovibrissa-mediated) sampling.

Long-distance sampling used the macrovibrissae because i) microvibrissae did not reach the surface, ii) trimming microvibrissae did not impair discrimination, and iii) trimming macrovibrissae reduced performance to chance (**Fig. 4**). In addition, visual cues were not available, and olfactory controls showed that neither intrinsic olfactory cues in sandpapers nor deposited olfactory cues in sandpapers or grooved aluminum surfaces were used for discrimination (**Fig. 6**). Rats failed to discriminate between two identical sandpapers (P150 vs. P150 or P1500 vs. P1500), confirming that animals were not discriminating based on extraneous stimuli in the training environment, e.g. motor rotation sounds (**Fig. 5**).

In contrast, short-distance discrimination of smooth-grooved surfaces did not depend on macrovibrissae, and surprisingly was only modestly dependent on microvibrissae (**Fig. 4**). Because olfactory and visual cues were ruled out, we infer that short-distance discrimination primarily involves tactile input from direct skin/fur contact. Short-distance discrimination of smooth-grooved surfaces therefore differs surprisingly from short-distance sensation of object shape, which depends absolutely on microvibrissae [4]. Functionally, short-distance discrimination was more accurate than long-distance discrimination for smooth-grooved judgments, and for one rat performing sandpaper judgments (**Fig. 3**). This suggests that microvibrissae or skin/fur cues can provide more salient texture information than coarsely spaced macrovibrissae [4], [25]. Nonetheless, long-distance macrovibrissa-based texture discrimination was robust, and significantly above chance for all 5 rats studied here. Rats intermixed short- and long-distance sampling if permitted, and the fraction of short-distance trials increased when macrovibrissae were trimmed (**Fig. 4A**). This indicates that sampling strategy is dynamically optimized, akin to the multiple whisking strategies used for spatial localization [37], [38], [39].

Many prior studies report texture discrimination using the whiskers, but none have rigorously distinguished between macro- and microvibrissae-based strategies. Most studies used a gap to promote macrovibrissa use [16], [17], [18], [19], [20], [22], [40], but microvibrissae were either not monitored [16], [19], [20], [40] or also contacted the surface [18], [21]. Thus, microvibrissae could have aided texture sensation in these prior studies.

### Psychometric function for macrovibrissa-based sandpaper texture discrimination

A major goal of this study was to measure a psychometric curve for macrovibrissa-based texture discrimination, in order to provide a behavioral benchmark for neural models of texture coding. Comparison of psychometric and neurometric curves is a gold-standard approach to evaluating sensory codes [41], but has not yet been applied in whisker texture sensation. Commercial sandpapers are reasonable texture stimuli for constructing a psychometric curve, because they are uniform in two dimensions, have standardized mean grain sizes, and are readily available. The main drawback of sandpapers is the use of different grain materials, paper backings and glues to manufacture different roughnesses. This may lead to physical properties that do not vary smoothly from coarsest to finest sandpapers, and potential olfactory cues that could be used for discrimination. Despite these concerns, rats discriminated sandpapers in a graded manner that varied with differences in mean grain size (**Fig. 5**), and used whisker-based, not olfactory cues (**Figs. 4** **,** **6**).

We found that rats used long-distance sampling to discriminate surprisingly fine differences between sandpapers. Discrimination accuracy was reasonably well fit by a logistic sigmoid function of mean grain size difference (**Fig. 5**). Maximal discrimination performance for 2 rats was P150 vs. P180 (100 µm vs. 82 µm mean grain size). A third rat discriminated P150 vs. P400 (100 µm vs. 35 µm), but unfortunately finer distinctions were not tested in this animal. One rat discriminated P1500 vs. P800 (12.6 µm vs. 21.8 µm) but not P1500 vs. P1200 (12.6 µm vs. 15.3 µm). Thus, rats could distinguish surfaces that varied by as little as 10–20 µm mean grain size (**Fig. 5**). This substantially exceeds the best resolution reported previously for rats (sandpaper: 201 µm vs. 100 µm mean grain size [20], grooved surfaces: 1.00 vs. 1.06-mm groove spacing [18]) and mice (sandpaper: 190 µm vs. 50 µm mean grain size [40]). Comparison of the psychometric curve fits for rough vs. fine base sandpapers (Fig. 5B vs. 5D) suggests that rats can make fine discriminations relative to fine sandpaper (e.g., a 10 µm difference using a P1500 base sandpaper), but can only make coarser discriminations relative to a coarse sandpaper (P150), consistent with Weber's law. Rats were unable to discriminate P150 vs. P400 sandpaper when presented in reversed orientation, indicating that intrinsic olfactory cues were not used for discrimination (**Fig. 6**). Macrovibrissa trim reduced performance to chance, confirming that whiskers were used in this task (**Figs. 4** **,** **5C**).

The high resolution observed here may reflect the side-by-side placement of the two sandpaper samples, which allowed simultaneous, direct comparison. In contrast, prior studies presented two spatially separate textures, or only a single texture per trial, which requires comparison of tactile information about the current sample with a reference in working- or long-term memory [16], [17], [18], [19], [20], [22], [40]. However, it is not clear that rats readily perform sensory comparisons, but may instead memorize single stimuli. An additional explanation for the high acuity is the long training period in our animals prior to measurement of the psychometric curve (52–90 days). While we quantified discrimination relative to grain size difference, texture sensation may be determined by other physical properties (e.g., whisker-surface friction), in addition to or instead of grain size *per se*.

### Implications for neural coding of texture

Our results have implications for competing theories of neural coding of texture coding in the whisker system [23], [24]. The mean speed theory proposes that the relevant cue for texture is the mean speed of whisker micromotion calculated over the entire 100–300 ms epoch of surface whisking and encoded by mean firing rate in primary somatosensory cortex (S1)[22], [26]. The slip-stick theory proposes that the primary cues for texture are brief, high-velocity/high-acceleration whisker micromotions (slips and sticks) whose size and rate vary with texture, and which are encoded by transient spiking on the 20-ms time scale [26], [28], [30]. Mean speed and S1 firing rate vary between very rough and very smooth surfaces (e.g., P150 vs. smooth plastic) [22], but not between P150, P400, P800 and P1200 sandpapers [26], [30], suggesting that the mean speed model may explain detection of large, but not fine, texture differences. The rate of high-acceleration whisker slips differs between P150, P400, P800 and P1200 sandpapers [28], and firing correlations between pairs of S1 neurons on the 20-ms time scale differs between P150 and P1200, but not between P150 and P400 sandpapers [30]. Thus, unless significantly more texture information is present in larger neuronal populations, the slip-stick model can also only explain detection of relatively large texture differences.

We tested one rat's ability to perform difficult texture discrimination using only a single D1 whisker on each side (Fig. 5C). This manipulation was designed to test the differential resonance theory that texture is encoded by relative amplitude of sustained vibrations across different-length whiskers within a row [42]. Though an n = 1 experiment must be interpreted with extreme caution, this rat was able to discriminate P150 vs. P280 surfaces with the single whisker, suggesting that comparison of vibrations across different length whiskers was not required for texture discrimination in this task.

The present results show that behavioral discrimination using the macrovibrissae is substantially better than previously published discrimination limits, or than predicted by neural recordings of mean firing rate and slip-induced firing correlations. This suggests that additional cues besides mean speed and whisker slips may mediate discrimination of the finest texture differences, similar to the multiplex coding scheme for tactile detection in primate fingertips [43]. Alternatively, mean speed and whisker slips may be more sensitive cues for texture during performance of our discrimination task than previously measured in anesthetized, artificially whisking animals [26] or awake, actively whisking but non-discriminating animals [28], [30]. Distinguishing these possibilities and identifying additional texture coding strategies will require simultaneous neural recording and imaging of whisker micromotion during texture discrimination behavior.
